# Editorial: Anxiety, burnout, and stress among healthcare professionals

**DOI:** 10.3389/fpsyg.2023.1348250

**Published:** 2024-01-11

**Authors:** Vasfiye Bayram Deger

**Affiliations:** Nursing Department, Faculty of Health Sciences, Mardin Artuklu University, Mardin, Türkiye

**Keywords:** anxiety, burnout, stress, psychological, healthcare professionals

The technology, informatics and social fields are experiencing continuous changes and developments day by day. Such changes and developments influence human life and expand the fields of research. One of these fields is the world of work life. Work life is an area where people spend a significant part of their lives by spending time and effort. The willingness of employees to make quality use of their time and labor significantly affects the efficiency obtained as a result of their work (Burton, 2010). The nature of the work requirements and the quantity of communication with other people in the workplace create challenging situations for employees. In this context, when it comes to health in work life, health workers are seen as an important sample group in terms of researching variables in work life, since their field requires qualified labor force, is vital and is a profession that constantly involves face-to-face interaction with people. The health sector is one of the sectors where employees have the most difficulties due to various factors. The health sector differs from other working environments due to the difficulty of serving patients with severe stress and the fact that employees in this sector often face stressful situations in their daily working environment.

Stress, anxiety and burnout experienced by physicians, nurses and allied health personnel who are in direct contact with patients affect both their work performance and health status thus decreasing their quality of life. Anxiety that arises in healthcare professionals during or as a result of crisis intervention can impair their mental reasoning and abstract thinking skills, leading to a lack of attention and coordination. Various emotions such as fear and anxiety can affect problem-solving performances. Decreased problem solving ability may lead to a decrease in the effectiveness of services provided to protect the health of individuals and society and to facilitate living conditions (Çelmece and Menekay, 2020).

Anxiety in health care workers can lead to occupational injuries and illnesses; it particularly affects physical, social and psychological health negatively. Burnout is the psychological response of health workers. A significant relationship exists between anxiety and burnout syndrome and anxiety may predispose to burnout syndrome. Healthcare workers are exposed to burnout as a result of psychological and behavioral actions due to stress and pressure caused by intense communication and the nature of the work area. The state of anxiety and burnout undoubtedly carries the risk of bringing about a decrease in the functionality of healthcare workers.

In addition, inadequacies in health services and unbalanced distribution of health workers also create a sense of stress on workers. Factors such as improper working conditions, insufficient personnel, lack of medical equipment and supply problems can negatively affect the mental health of healthcare workers. Stress and exhaustion based on this working environment can lead to mental symptoms such as depression, anxiety and feelings of helplessness. In addition, physiological symptoms such as headaches, muscle tension and insomnia may also occur. Healthcare workers may experience sleep disturbances due to working in shifts and work stress, which can negatively affect their work performance. Healthcare workers are often witness to the harsh realities of life. This is especially true for those who have to witness traumatic events. Healthcare workers working in emergency departments and intensive care units are often confronted with death, suffering and emergencies. This can lead to a higher incidence of illnesses such as acute stress disorder, post-traumatic stress disorder, depression, psychosomatic disorders and substance use disorders in healthcare workers.

The health system is one of the institutions operating under the most challenging conditions, especially in epidemics or extraordinary situations such as the pandemic we have recently experienced, which affected the whole world and caused deaths. The mental health of healthcare teams, who undertake an intense social and work burden, especially during periods of extraordinary situations that affect the society socioeconomically and psychologically, is also affected by this situation. There are a large number of studies in the literature on the subject, and there are many studies that focus on the psychological risks of healthcare workers, especially in relation to epidemics, identifying high levels of anxiety, depression, stress and burnout (Cocco et al., 2002; Wong et al., 2005; Zhang et al., 2018). Moreover, there were even disparities among health workers, with health workers who were on the front lines of diagnosing, treating and caring for their patients during the COVID-19 pandemic reporting more severe symptoms of anxiety, depression and stress than those who were not on the front lines (Kang et al., 2020; Lai et al., 2020). In the early stages of an epidemiological crisis, symptoms such as anxiety and fear increase immediately, but decrease rapidly in the later stages, while depression and post-traumatic stress symptoms persist over time (Wu et al., 2009). Cai et al. (2020) surveyed 534 doctors, nurses and primary care providers in Hubei province and concluded that the stress levels of healthcare workers were extremely high during the COVID-19 pandemic. Another study conducted in Turkey found that 38% of nurses working in emergency services experienced stress. According to the same study, it was revealed that nurses who experienced stress regretted their choice of profession and considered quitting or leaving their jobs (Yasal and Partlak, 2019).

In light of this information, some precautions should be taken for healthier health workers and thus better health care services, where the risk of error is minimized and care is better. More healthcare workers can be employed in institutions to reduce the pressure and workload. During the pandemic, one of the most challenging issue was access to appropriate protective equipment. Personal protective equipment can be provided for health professionals. Working hours can be rescheduled by planning the rest needs of healthcare professionals and creating working and resting environments that will ensure that the risk of infection as well as other risk factors arising from lack of sleep and fatigue are controlled.

In conclusion, some steps that may be taken to resolve this issue are likely to have a positive impact on public health. Healthcare institutions should prioritize the psychological health of their employees and create a supportive working environment. Institutional policies should include measures such as a less stressful working environment, supportive services for managing employees' workloads and flexibility for work balance, and they can provide regular mental health trainings for health workers. These trainings can provide the necessary tools to overcome work stress, prevent burnout and maintain psychological health. Establishing support groups for health workers can empower workers to interact with each other, share work stress and receive support.

## Author contributions

VB: Writing – original draft.
